# Astrocytoma Mimicking Herpetic Meningoencephalitis: The Role of Non-Invasive Multimodal Monitoring in Neurointensivism

**DOI:** 10.3390/neurolint15040090

**Published:** 2023-11-29

**Authors:** Uri Adrian Prync Flato, Barbara Cristina de Abreu Pereira, Fernando Alvares Costa, Marcos Cairo Vilela, Gustavo Frigieri, Nilton José Fernandes Cavalcante, Samantha Longhi Simões de Almeida

**Affiliations:** 1 Hospital Samaritano Higienópolis—Américas Serviços Médicos, São Paulo 01232-010, Brazil; barbara.pereira@samaritano.com.br (B.C.d.A.P.); fernando.costa@samaritano.com.br (F.A.C.); marcos.vilela@samaritano.com.br (M.C.V.); ncavalcante2009@hotmail.com (N.J.F.C.); samantha.almeida@samaritano.com.br (S.L.S.d.A.); 2 Hospital Israelita Albert Einstein, Faculdade Israelita de Ciências da Saúde Albert Einstein, São Paulo 05652-900, Brazil; 3 Medical Investigation Laboratory 62, School of Medicine, University of São Paulo, São Paulo 01246-000, Brazil; gustavo.frigieri@brain4.care

**Keywords:** viral encephalitis, astrocytoma, multimodal, monitoring, intensive care unit

## Abstract

Neuromonitoring is a critical tool for emergency rooms and intensive care units to promptly identify and treat brain injuries. The case report of a patient with status epilepticus necessitating orotracheal intubation and intravenous lorazepam administration is presented. A pattern of epileptiform activity was detected in the left temporal region, and intravenous Acyclovir was administered based on the diagnostic hypothesis of herpetic meningoencephalitis. The neurointensivist opted for multimodal non-invasive bedside neuromonitoring due to the complexity of the patient’s condition. A Brain4care (B4C) non-invasive intracranial compliance monitor was utilized alongside the assessment of an optic nerve sheath diameter (ONSD) and transcranial Doppler (TCD). Based on the collected data, a diagnosis of intracranial hypertension (ICH) was made and a treatment plan was developed. After the neurosurgery team’s evaluation, a stereotaxic biopsy of the temporal lesion revealed a grade 2 diffuse astrocytoma, and an urgent total resection was performed. Research suggests that monitoring patients in a dedicated neurologic intensive care unit (Neuro ICU) can lead to improved outcomes and shorter hospital stays. In addition to being useful for patients with a primary brain injury, neuromonitoring may also be advantageous for those at risk of cerebral hemodynamic impairment. Lastly, it is essential to note that neuromonitoring technologies are non-invasive, less expensive, safe, and bedside-accessible approaches with significant diagnostic and monitoring potential for patients at risk of brain abnormalities. Multimodal neuromonitoring is a vital tool in critical care units for the identification and management of acute brain trauma as well as for patients at risk of cerebral hemodynamic impairment.

## 1. Introduction

Inflammation in the central nervous system can be a symptom of the disorder referred to as viral encephalitis [1], which is typically caused by illnesses resulting from a variety of pathogens. There is much variation in the clinical presentation of viral encephalitis [2], which can range from relatively minor symptoms such as headache and fever to more severe neurological abnormalities [3]. The propensity of viral encephalitis for mimicking other neurological disorders, most notably glioma, is one of the disease’s most intriguing aspects. Gliomas [4] are a specific kind of brain tumor that develops from glial cells and frequently exhibits symptoms like those of encephalitis, such as seizures, cognitive impairment, and focal neurological abnormalities. The similarities in the clinical presentation and radiological findings between viral encephalitis and glioma present substantial diagnostic challenges for healthcare professionals. An incorrect diagnosis can result in inappropriate therapy, which can significantly deteriorate the patient’s condition. Even though sophisticated imaging methods like magnetic resonance spectroscopy (MRS) and positron emission tomography (PET) have been utilized to distinguish between the two illnesses, the diagnostic conundrum still exists [5]. In the treatment of viral encephalitis as well as glioma, intracranial hypertension (ICH) is another important consideration because of its deleterious effects, including brain herniation [6] and even death. In the past, procedures such as intraventricular [7] or intraparenchymal monitoring, both of which are invasive, were the standard way to detect and monitor intracranial pressure (ICP). On the other hand, these procedures are not without danger, such as the possibility of infection or bleeding. In recent years, there has been an increasing interest in creating non-invasive procedures for monitoring [8]. Emerging non-invasive approaches, such as transcranial Doppler ultrasonography and measuring the diameter of the optic nerve sheath [9] using ultrasound, are some examples of potential bedside technologies. When compared with their more invasive equivalents, these technologies are easier to replicate and pose a lower risk to the patient. In order to provide the best possible care for patients, an integrated strategy is necessary, due to the possible difficulty of distinguishing viral encephalitis from glioma and the difficult and challenging task of monitoring ICP. Innovative imaging modalities may provide hints for a differential diagnosis, while non-invasive methods may enable continuous monitoring of intracranial pressure to guide therapeutic actions. This case report describes the initial presentation of meningoencephalitis mimicking an astrocytoma in the central nervous system and the importance of a multimodal approach using non-invasive techniques to monitor ICH [10,11] and improve patient outcomes. Multimodal non-invasive techniques for monitoring ICH encompass a diverse array of methods (ultrasound, skull strain sensors) designed to assess elevated intracranial pressure (ICP) without the need for placement of an intraventricular catheter (gold standard) [12].

## 2. Case Presentation

A 61-year-old Latin female developed acute mental disorientation and dysarthria at home lasting 3 h, despite no previous history of disease or treatment. On approach to the hospital, the patient experienced an 8 min generalized tonic–clonic seizure (TCS), prompting orotracheal intubation and sedation with intravenous lorazepam in accordance with institutional procedures. On admission, a head CT scan revealed a hypodense pattern in the left temporal lobe and cerebrospinal fluid with the presence of 12 cells, lymphocyte predominance, 22 red blood cells, proteins 68, glucose 64, lactate 16, and pan-herpes PCR negative, prompting transfer to the ICU (Table 1).

**Figure 1:**
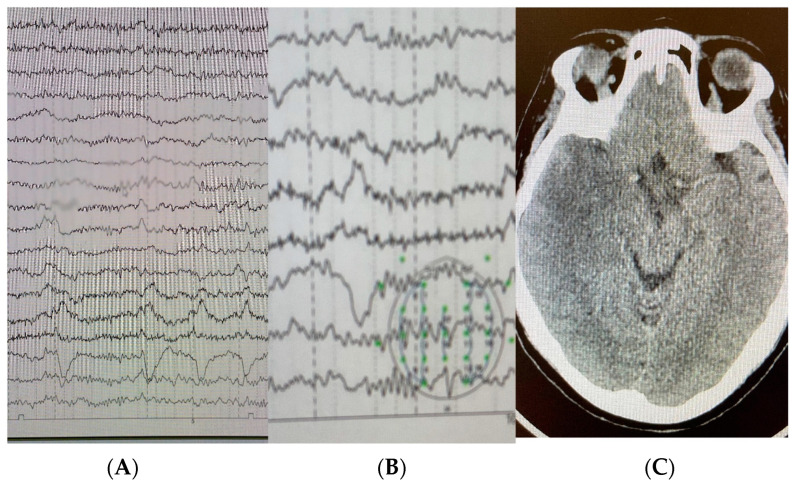
(**A**) Electroencephalogram 12 channels. (**B**) Epileptiform activity in the temporal regions. (**C**) Hypodensity area in the left temporal central nervous system.

Upon electroencephalogram admission (Figure 1), elytriform activity was detected in the left temporal region, and intravenous aciclovir was administered based on the diagnostic hypothesis of herpetic meningoencephalitis.

**Figure 2:**
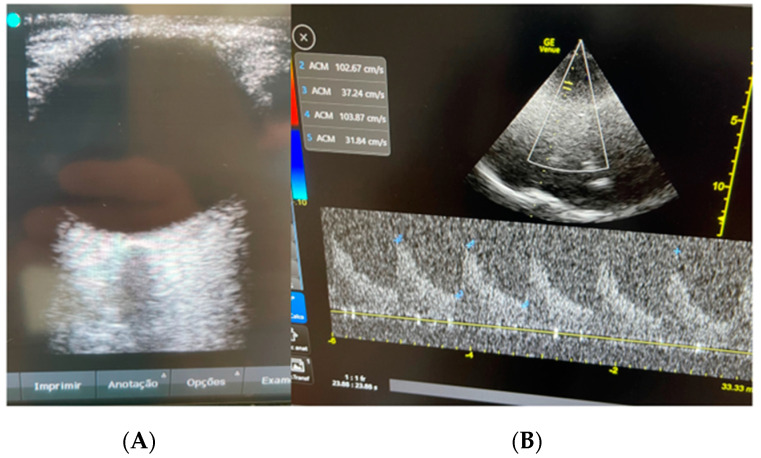
(**A**) Optic nerve sheath diameter 5.6 mm. (**B**) Mean flow velocity of the middle cerebral artery 59 cm/s. Pulsatility index of 1.2. Resistance Index 0.7.

**Figure 3:**
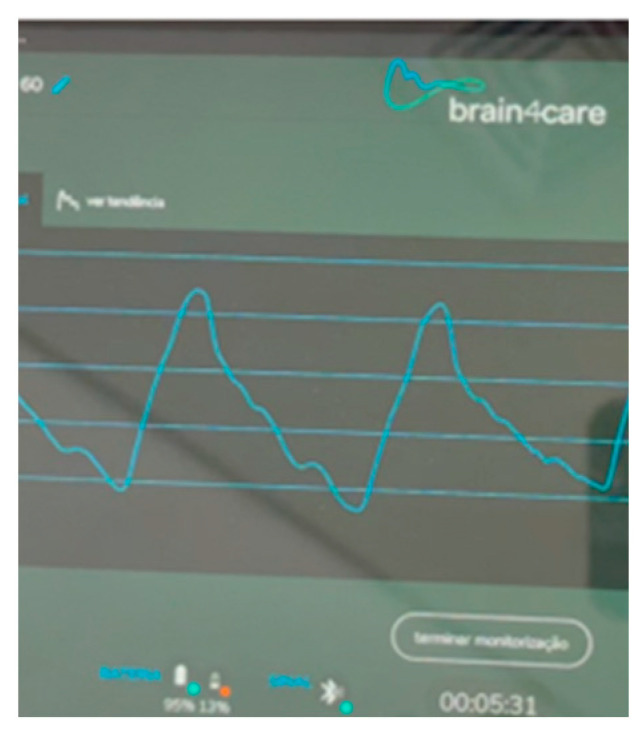
Brain4care device waveform in real time demonstrating a low cerebral complacency, P2 wave > P1 wave.

Sedation was weaned after 48 h of mechanical ventilation and rigorous neuroprotective treatments (strict control of temperature and blood glucose, avoiding systemic arterial hypotension, protective mechanical ventilation, and adequate analgesia). Following the sedation weaning, the patient experienced a new episode of TCS, and multimodal non-invasive bedside neuro-monitoring was chosen due to their condition’s complexity and the impossibility of immediate transfer to the radiology sector. A Brain4care (B4C) non-invasive intracranial compliance monitor along with measurement of the optic nerve sheath (ONSD) and transcranial Doppler (TCD) were employed (Figure 2 and Figure 3) to identify complications such as ICH.

**Figure 4:**
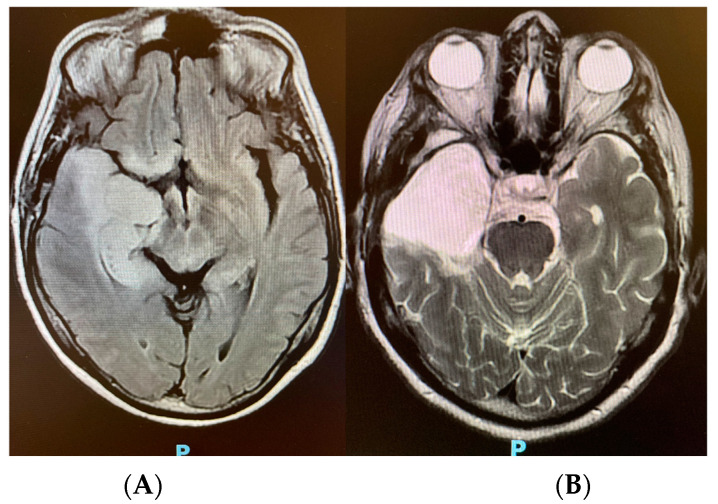
(**A**) Brain MRI demonstrating left temporal lesion with compression of lateral ventricles. (**B**) Post-surgical status after resection of brain tumor.

Based on the gathered data, ICH was diagnosed and bedside emergency interventions such as sedation, mannitol, and hyperventilation were initiated to improve intracranial compliance and reduce morbidity. Magnetic resonance imaging (MRI) was performed, revealing signs of a deviation of the medial line (4.0 mm) and temporal pole image, with an expansive effect characterized by deletion of the grooves and regional fissures and compression of the ipsilateral lateral ventricle, as well as the possibility of infectious limbic encephalitis, which is primarily caused by herpes virus type 1 (HSV-1). After 5 days of hospitalization and ventilatory weaning, the neurosurgical team requested a second MRI and ran a new pan-herpes PCR that proved negative. A stereotaxic biopsy of the temporal lesion was performed to explore a differential diagnosis. The intraoperative histopathology revealed a grade 2 diffuse astrocytoma, and a complete resection was performed immediately (Figure 4).

## 3. Discussion

Status epilepticus [13,14] is a neurological emergency caused by a variety of intracranial and extracranial disorders, including infections and tumors. Early detection and treatment of SE improves outcomes and reduces long-term complications [15]. Patients with SE and epileptogenic activity in the temporal area, accompanied by a hypodense image of the temporal region on a head CT scan and a pattern of predominance of lymphocytes in the cerebrospinal fluid, should be evaluated for herpetic encephalitis (HE) [16] and treated immediately with antiretroviral medication. Viral meningoencephalitis-associated complications [17] in the central nervous system (CNS) can lead to vasculitis and thrombotic events. HE has been shown to predominantly affect the medial temporal lobe [18], and sometimes the frontal lobes and the cingulate gyrus, while the basal ganglia are normally spared. HE has a bimodal distribution, with individuals younger than 20 and older than 50 years being equally affected. ICH [19] is a condition marked by elevated intracranial pressure and can be caused by many underlying diseases, with symptoms such as headaches, visual deficits, and mental state changes. Identification and treatment of the underlying cause, as well as the use of drugs to lower ICH, are common components of its management [20]. In refractory cases, surgical intervention (decompressive craniectomy) may be required to alleviate head pressure [21,22].

Transcranial Doppler (TCD) [23,24] may be used to indirectly evaluate ICH. As intracranial pressure rises, cerebral blood arteries may become constricted, resulting in a reduction in blood flow velocity. TCD can detect these changes in blood flow velocity and estimate intracranial pressure. The pulsatility index (PI) [25] is a TCD measurement that has been used to assess intracranial pressure (PI). PI is a measure of the pulsatility of the waveform of cerebral blood flow and is computed as the difference between the peak systolic and end diastolic velocities divided by the mean velocity. When intracranial pressure rises, cerebral blood vessels become less flexible, resulting in a rise in PI. TCD has been shown to be a reliable [26] and non-invasive method for monitoring changes in intracranial pressure in individuals with a variety of neurological disorders [27]. TCD cannot directly measure intracranial pressure but is instead used to estimate intracranial pressure. It must be interpreted with care and in combination with other clinical and radiological examinations.

The pulsatility index (PI) is a standard TCD measurement of flow resistance. It is calculated by subtracting end diastolic velocity (EDV) from peak systolic velocity (PSV) and dividing the result by the mean flow velocity (Vm) (MFV). A PI value higher than 1.2 suggests substantial blood flow resistance. In clinical settings, a high pulsatility index in the middle cerebral artery (MCA) has been linked to stroke progression [28] and neurological impairment following acute cerebral infarction [29]. In addition to the pulsatility index (PI), the resistive index (RI) [30] is often used to quantify the resistance of a pulsatile vascular system. The RI is determined by subtracting the EDV from the PSV and dividing the resulting number by the PSV. In clinical practice, the pulsatility index is a reliable TCD metric used to evaluate intracranial pressure and blood flow alterations in a variety of clinical scenarios, including stroke, traumatic brain injury, and hyperbilirubinemia in newborns.

Ultrasound of the optic nerve sheath diameter (ONSD) [31] has evolved in recent years as a non-invasive method for assessing intracranial pressure (ICP) or identifying ICH. It is a safe bedside procedure that can be performed in real time, is repeatable and very inexpensive, and has no radiation risks [32]. The ONSD varies with the cerebrospinal fluid (CSF) pressure owing to the layer of subarachnoid space between the optic nerve and its sheath, which swells in response to increased intracranial pressure. Studies have shown a link between elevated intracranial pressure and optic nerve sheath diameters higher than 5 mm, as measured 3 mm posterior to the retina [33,34]. A comprehensive review and meta-analysis have shown that an ONSD greater than 5.0 to 5.70 mm is associated with ICP of more than 20 mm Hg [35]. The upper-limit maximum ONSD threshold varies from 4.8 to 6.2 mm when measured 3 mm from the globe, while the average maximum ONSD is 5.8 mm when measured 8 mm from the globe on a CT scan. Transorbital ONSD measurement is an emerging approach in the detection and monitoring of ICH. Increasingly, ultrasound examination of the ONSD is utilized as a marker to identify elevated ICP. A non-invasive ONSD ultrasonography may be used to assess intracranial pressure or identify ICH, and the relationship between the ONSD and intracranial pressure results is well established.

Brain4care [36,37,38] is a non-invasive point-of-care technology that detects micrometric changes in the skull volume. Monitoring these small deformations reveals a pulse morphology similar to the intracranial pressure waveform (ICPwf), an important metric related to intracranial compliance and to the mean value of intracranial pressure. The Brain4care report showed two parameters, the P2/P1 ratio (P2/P1) and time to peak (TTP). P2/P1 is the ratio between the amplitude of the P2 pulse and that of the P1 pulse. Values above 1.0 indicate that the P2 component is higher than P1, characterizing a reduction in intracranial compliance, and values greater than 1.2 suggest ICH. TTP is the direct measurement of the normalized time between the beginning of the pulse and its highest point; values above 0.25 indicate changes in morphology and impairment of intracranial compliance. Although many studies [39] have shown that ICPwf analysis helps in predicting increased intracranial pressure and may be an important alarm, Brain4care was the first quantitative and automated method of monitoring intracranial compliance and evaluating ICH to be incorporated into our clinical practice [40]. Neuromonitoring methods have evolved into an indispensable tool in emergency departments and intensive care units for the early diagnosis and treatment of traumatic brain injuries. Studies have indicated that monitoring patients in a specialized neurologic intensive care unit (Neuro ICU) may result in better outcomes and shorter hospital stays [41]. In addition to being advantageous for patients with a main brain injury, neuromonitoring may be beneficial for people at risk of cerebral hemodynamic impairment who do not have a primary brain injury [42]. In the neonatal intensive care unit (NICU), typical neuromonitoring methods include amplitude-integrated electroencephalography and multichannel conventional electroencephalography [43].

Non-invasive monitoring multimodalities for assessing ICH, while advantageous in reducing the risks associated with direct intracranial interventions, present several inherent limitations. First, these techniques offer indirect indicators of ICP rather than direct measurements, potentially leading to inaccuracies. The results can exhibit significant inter-individual variability, as seen in measurements of the ONSD, making it challenging to establish universally applicable thresholds. Moreover, certain modalities such as TCD are operator-dependent [44,45,46], with outcomes that vary based on the practitioner’s expertise. External factors, like changes in the carbon dioxide levels in TCD [47] or the intraocular pressure of the ONSD, can also influence interpretations. Additionally, the lack of standardization across some of these techniques [48] can result in interpretation discrepancies [49]. Consequently, while non-invasive methods provide valuable insights, they should be interpreted cautiously and, ideally, in conjunction with other clinical assessment data [50].

## 4. Conclusions

The presentation of astrocytoma that resembles herpetic meningoencephalitis poses a distinct problem in the field of neurointensivism in terms of both diagnosis and treatment. The overlapping clinical and radiological characteristics of both illnesses can result in misdiagnosis and incorrect therapy. Non-invasive multimodal monitoring approaches, including TCD, B4C, and ONSD, have been recognized as aids in the management of these intricate situations. These methodologies enable the monitoring of ICP and cerebral perfusion in real time, facilitating prompt treatment and mitigating the potential hazards associated with invasive techniques. The incorporation of non-invasive monitoring techniques into neurointensive care regimens will enhance diagnostic precision and patient outcomes.

## Figures and Tables

**Table 1:** Comprehensive summary of the patient’s clinical presentation and treatment. CSF: cerebral spine fluid; ONSD: optic nerve sheath diameter; TCD: transcranial Doppler; B4C: Brain4care.

Date	CSF	ONSD	TCD	B4C
D 0	Pan-herpes negative			
Day 2		5.6 mm	MCA 59 cm/s; PI 1.2; RI 0.7	P2 > P1
Day 2			MCA 65 cm/s; PI 1.0; RI 0.5	P1 > P2
Day 5	Pan-herpes negative			

## Data Availability

Data are contained within the article.
